# Superficial siderosis as a rare cause of visual and auditory pseudohallucinations: a case report

**DOI:** 10.1186/s13256-021-02902-6

**Published:** 2021-07-10

**Authors:** Tobias Braun, Maxime Viard, Tobias Struffert, Omar AlhajOmar, Mesut Yeniguen, Martin Juenemann

**Affiliations:** 1 Department of Neurology, University Hospital Giessen and Marburg, Justus Liebig University, Klinikstrasse 33, 35392 Giessen, Germany; 2 Department of Neuroradiology, University Hospital Giessen and Marburg, Klinikstrasse 33, 35392 Giessen, Germany

**Keywords:** Superficial siderosis, Pseudohallucinations, Case report

## Abstract

**Background:**

Superficial siderosis is a rare disease involving hemosiderin deposits on the surface of brain or spinal cord that are thought to cause clinical symptoms, which usually consist of cranial nerve dysfunction, cerebellar ataxia, or myelopathy. Pseudohallucinations have been described as the patient being aware of the nonreality of hallucination-like phenomena. Data on pseudohallucinations of cerebral somatic origin are sparse. We present a case of auditory and visual pseudohallucinations due to superficial siderosis. Siderosis was diagnosed using cerebrospinal fluid analysis and magnetic resonance imaging as part of the clinical routine for newly emerged psychiatric symptoms.

**Case presentation:**

An 84-year-old white/european female presented to our hospital with no prior history of psychiatric or neurological disease and no history of trauma. She reported seeing things and hearing voices singing to her for some days. She was aware these phenomena were not real (pseudohallucinations). On examination, no relevant abnormalities were detected. Cerebrospinal fluid analysis showed elevated ferritin. Magnetic resonance imaging with susceptibility-weighted sequences revealed diffuse superficial siderosis in several parts of the brain, among other occipital and temporal gyri. The pseudohallucinations resolved with a risperidone regime. The patient was treated with rivaroxaban because of atrial fibrillation. Potentially elevating the risk of further hemorrhage, this therapy was discontinued, and an atrial appendage occlusion device was implanted.

**Conclusion:**

We report the first case of pseudohallucinations in superficial siderosis. The risk of missing this diagnosis can be reduced by applying a standardized diagnostic pathway for patients presenting with the first episode of psychiatric symptoms. Somatic and potentially treatable causes should not be missed because they might lead to unnecessary treatments, stigmatization, and legal restrictions of self-determination, especially for elderly people.

## Background

Superficial siderosis is a rare disease with recurrent or persistent hemorrhage in the subarachnoid space. Hemosiderin is deposited on brain or spinal cord surfaces and is thought to cause clinical symptoms that usually consist of cranial nerve dysfunction, cerebellar ataxia, or myelopathy [1].

Pseudohallucinations have been described as the patient being aware of the nonreality of hallucination-like phenomena [2]. Data on pseudohallucinations of cerebral somatic origin are sparse. A known cause of visual pseudohallucinations in elderly patients is severely impaired vision (that is, due to ophthalmologic disease such as Charles Bonnet syndrome). Patients can also develop this syndrome following occipital cerebral ischemic stroke or cardiac surgery [2].

We present the case of an 84-year-old female who presented with auditory and visual pseudohallucinations due to superficial siderosis.

## Case presentation

An 84-year-old white/european female presented to the hospital’s emergency department complaining of seeing people who were not present in the room and of hearing voices singing to her. The symptoms started acutely several days prior. She was aware that the phenomena were not real. It was her first ever episode. She did not have a history of neurological or psychiatric diseases. She had a medical history of ankylosing spondylitis, arterial hypertension, atrial fibrillation, chronic heart failure, and coronary heart disease. In the past, surgery was performed on her right eye because of glaucoma, and she underwent implantation of a biological heart valve in the aortic position. Her prescribed medication comprised rivaroxaban (in adequate dose), spironolactone, torasemide, bisoprolol, irbesartan, amlodipine, simvastatin, ezetimibe, and brinzolamide/timolol eye drops, as well as moisturizing eye drops.

Initial physical examination revealed anisocoria (right pupil larger than the left) due to the operation. There were no neurological deficits. Apart from the pseudohallucinations, there were no additional psychopathological findings. She appeared to be in good medical condition. Apart from mild anemia, no other abnormalities appeared in the laboratory test of blood and urine.

Initial computed tomography (CT) of the head did not provide an explanation for the pseudohallucinations. Electroencephalography revealed a discontinuous slowing in both parietal regions without epileptiform patterns. Thorough neuropsychological testing (CERAD battery (Consortium to Establish a Registry for Alzheimer's Disease), MMSE (Mini-Mental State Examination), and Brief Test of Attention) revealed normal cognitive function with fluctuating concentration, which was attributed to distraction due to the misperceptions. There were no signs of dementia or schizophrenia. An ophthalmological referral did not provide new explanations.

Lumbar puncture (after pausing rivaroxaban) revealed xanthochrome cerebrospinal fluid (CSF) with elevated leucocytes (8/µl; normal: <5/µl), heightened protein (0.754 g/l; normal: <0.45 g/l), and increased ferritin (129 ng/ml; normal: <15 ng/ml). Testing for antineuronal antibodies in serum and CSF, as well as microbiological and virological testing of the CSF, did not reveal abnormalities. CT angiography did not detect a source of a potential subarachnoidal hemorrhage that was suspected owing to the elevated ferritin. Because the leukocyte count in the CSF was elevated, we started the patient on intravenous aciclovir, which was omitted after virological test of CSF found no viral Desoxyribonucleic acid (DNA).

**Fig. 1:**
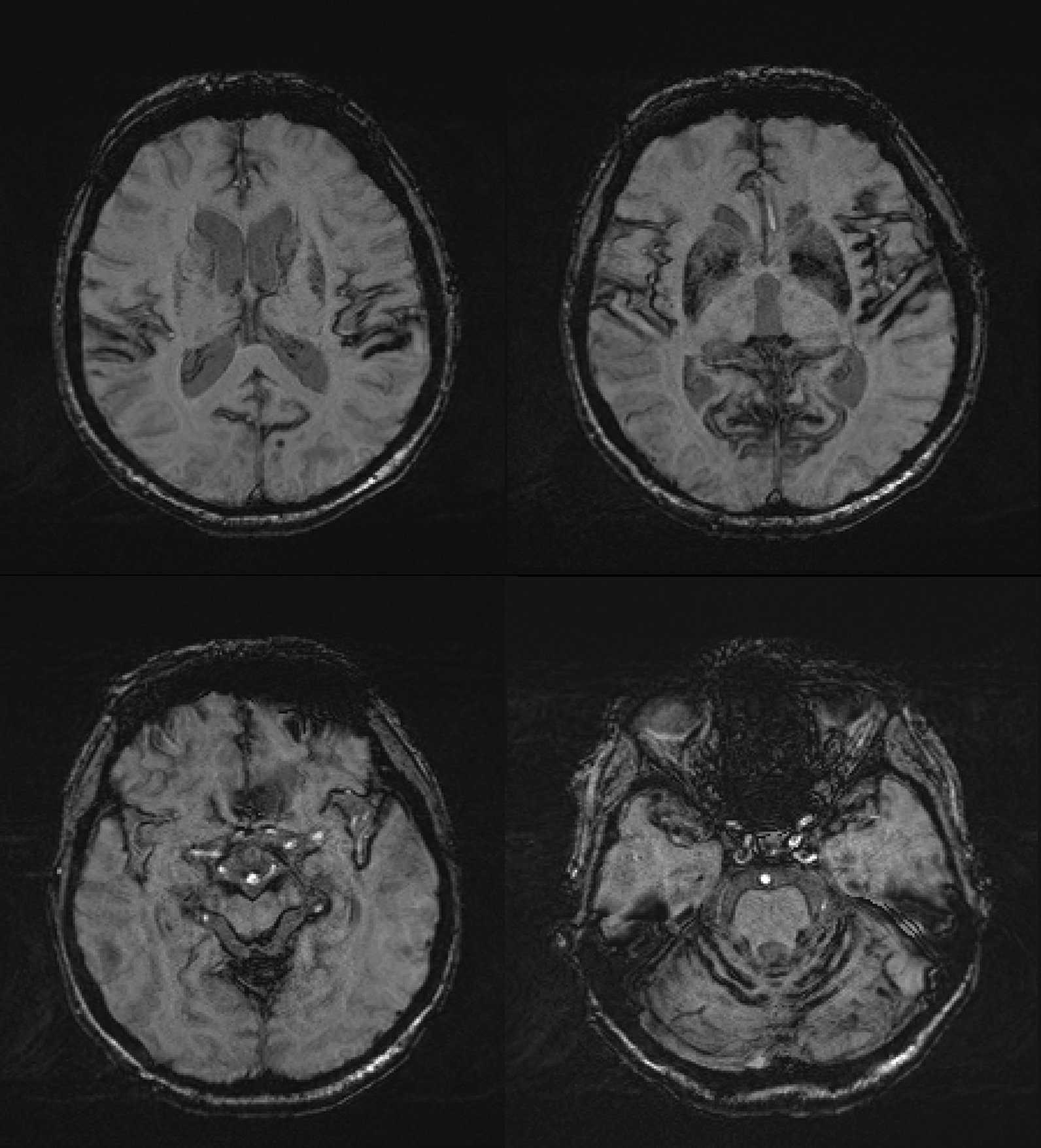
Axial susceptibility-weighted imaging sequences of the patient with bitemporal, bi-insular, bi-occipital, perimesencephal, peripontine, and cerebellar hemosiderin depositions

Magnetic resonance imaging (MRI) of the brain with susceptibility-weighted sequences finally showed diffuse bilateral superficial siderosis with bitemporal, bi-insular, and cerebellar emphasis (Fig. 1). No overt reason could be determined for this finding. We were unable to perform conventional angiography because of severe arteriosclerosis, elongation, and tortuosity of aorta and supra-aortic arteries. MRI of the spine did show hemosiderin remnants on the height of the first lumbar vertebra and the ankylosing spondylitis.

After prescribing risperidon (1.5 mg daily), the pseudohallucinations resolved during the stay in hospital. Because of elevated ferritin and the superficial siderosis, we did not continue rivaroxaban after the lumbar puncture. Nevertheless, protection from thromboembolic events due to atrial fibrillation was necessary. Therefore, we discussed implantation of an atrial appendage occlusion device with the cardiology department. Because dual antiplatelet therapy would necessitate implantation and because we were unable to rule out active bleeding, we planned to discharge the patient and readmit her for MRI of the brain 2 weeks later. The patient was put on enoxaparin for thrombosis prophylaxis because full anticoagulation was not possible owing to potential bleeding. Risperidone could be reduced to 0.5 mg daily. The new MRI showed the siderosis had not progressed, and the patient did not report the reappearance of visual or auditory pseudohallucinations. The patient was started on 100 mg of aspirin daily and 75 mg of clopidogrel daily (loading dosage 300 mg), and a left-atrial occluder was implanted without complications.

Over time, the patient was not readmitted to our hospital. During the last contact for consent for publication, no new symptoms were reported.

## Discussion and conclusion

Superficial siderosis of the brain is a rare disease of the central nervous system. Due to intermittent or ongoing hemorrhage, hemosiderin deposits in the spinal cord and the subpial layers of the brain. A common risk factor is prior history of trauma or intradural cranial surgery. Patients usually present with slowly progressive cranial nerve dysfunction, ataxia, or sensineural hearing loss [3]. A coincidence with Alzheimer’s disease and amyloid angiopathy has been described [4, 5]. Ferritin, which could be detected in our patient’s CSF, is synthesized by the brain as a reaction to contact with hemoglobin. Siderosis can be readily diagnosed using MRI with T2- and susceptibility-weighted sequences. The diagnosis is usually missed on CT of the head [3]. If a source for the hemorrhage can be found, then the source is appropriately treated. Iron-chelating therapy using oral deferiprone is currently gaining attention as a treatment option [1].

Data on pseudohallucinations of cerebral somatic origin are sparse. A known cause of visual pseudohallucinations in elderly patients is severely impaired vision (for example, due to ophthalmologic disease such as Charles Bonnet syndrome). Patients can also develop this syndrome following occipital cerebral ischemic stroke or cardiac surgery [2].

We identified one case of superficial siderosis in a 68-year-old male with a history of head trauma 20 years prior to auditory and visual disturbances. However, these were true hallucinations, and the patient developed a delusional state [6]. Therefore, our study is the first to report pseudohallucinations in superficial siderosis as the only symptom.

If a patient presents to our emergency department with unprecedented psychiatric disease, then CT is usually performed after taking history and clinical examination. We then analyze CSF for acute changes and send a sample to a specialized laboratory for evaluation for antineuronal antibodies (for example, N-methyl-D-aspartate (NMDA) or CASPR2). If the patient is agitated and aggressive on presentation, then we usually refer the patient to a psychiatric ward and recommend electroencephalography and MRI of the brain when the patient is calm enough for the examinations. Otherwise, these patients are admitted to our neurological ward where both examinations are performed. We recommend this approach to identify cases such as the one described or for differential diagnosis of psychiatric symptoms due to other disease entities (for example, tumor or encephalitis). Somatic and potentially treatable causes should not be missed, and newly emerged symptoms should not be attributed to age or suspected onset of dementia, especially in the elderly. This could lead to unnecessary treatments and stigmatization and might lead to legal restrictions of self-determination.

We report the first case of pseudohallucinations in superficial siderosis. The risk of missing this diagnosis can be reduced by applying a standardized diagnostic pathway for patients presenting with the first episode of psychiatric symptoms. This is of great importance, especially for the elderly.

## Data Availability

Data sharing is not applicable to this article as no datasets were generated or analyzed during the current study.

## Abbreviations

CT: Computed tomography
CSF: Cerebrospinal fluid
MRI: Magnetic resonance imaging
CERAD: Consortium to Establish a Registry for Alzheimer's Disease
MMSE: Mini-Mental State Examination
